# Regression of metastatic melanoma by targeting cancer stem cells

**DOI:** 10.18632/oncotarget.437

**Published:** 2012-01-28

**Authors:** Max Schlaak, Patrick Schmidt, Christopher Bangard, Peter Kurschat, Cornelia Mauch, Hinrich Abken

**Affiliations:** ^1^ Department of Dermatology and Venerology, Skin Cancer Center at the Center for Integrated Oncology; ^2^ Tumorgenetics, Department I of Internal Medicine; ^3^ Institute for Radiological Diagnostics; ^4^ Center for Molecular Medicine Cologne, University of Cologne, D-50931 Cologne, Germany; ^+^ Present Address: Cancer Stem Cell Laboratory, École Polytechnique Fédérale de Lausanne, CH-1015 Lausanne, Switzerland

**Keywords:** cancer stem cell, melanoma, CD20, rituximab

## Abstract

Current therapeutic regimens attempt to eliminate all malignant cells of a melanoma lesion. Pre-clinical data, however, indicate that melanoma is maintained by a minor subset of cancer cells, which are characterized by CD20 expression. We attempted to eliminate those cells in a progressing, chemotherapy-refractory metastatic melanoma patient by lesional injections of the anti-CD20 therapeutic antibody rituximab and concomitant dacarbazine treatment, which was ineffective as monotherapy. Although the frequency of CD20^+^ melanoma cells within the tumor lesions was initially about 2% and the bulk of tumor cells did not express CD20, rituximab treatment produced lasting remission accompanied by a decline of the melanoma serum marker S-100 to physiological levels. Detailed in-depth-analyses revealed a switch of serum cytokines from a T helper-2 to a pro-inflammatory T helper-1 cell profile. Apart from B cell elimination and decline in gammaglobulin levels, no grade 3/4 toxicity related to treatment was observed. Data provide the first clinical evidence that targeting the minor subset of CD20^+^ “melanoma sustaining cells” produces regression of chemotherapy-refractory melanoma and highlight the potency of selective cancer cell targeting in the treatment of melanoma.

## INTRODUCTION

Current regimens in cancer therapy attempt to eliminate all malignant cells of a tumor lesion; the approach is based on the assumption that every cancer cell has equal malignant capacities. The contrary paradigm that an established tumor lesion is hierarchically organized is supported by the enormous cellular heterogeneity of tumor lesions with a minority of tumor cells, but not a random cancer cell, which populates the tumor cell mass, initiates tumor growth and drives progression [1]. Cancer initiating cells renew themselves, are more resistant to chemo- and radiotherapy, stay quiescent for long time, and drift to distant sites to initiate metastases and relapse after treatment [2]. For instance, metastatic relapse of melanoma can occur more than a decade after curative surgical treatment of the primary lesion; this phenomenon is thought to be due to the same cancer initiating cell that drives cancer progression.

Although cancer stem cells have been experimentally proven for a variety of solid tumors and leukemia by their functional capacities, they do not share a common marker. In the melanoma CD20 was first reported to be expressed on those cells [3]; other reports showed ABCB5 [4], CD271 [5] and other markers depending on the assay used to test for cancer stem capacities. These tumor-initiating cells may be variable in number and must not necessarily be rare in the case of melanomas [6, 7]. Moreover, melanoma cells exhibit a remarkable plasticity since isolated melanoma cells of different phenotypes can initiate new tumor lesions by asymmetric cell divisions when transplanted under appropriate conditions. Once established, however, a minor subset of melanoma cells seems to maintain tumor progression. A major implication thereof is that specific elimination of the minor side population with tumor progression capacities may be sufficient to shrink the tumor in the long term. The assumption is sustained by mathematical models implying that successful tumor therapy requires eradication of those stem cells to produce complete clinical response [8]. A strong rationale for selective cancer cell elimination in melanoma therapy was most recently provided by the observation that targeted elimination of the less than 2% subset of CD20^+^ melanoma cells in a transplantation model can lastingly eradicate the tumor lesion [9, 10]. While those pre-clinical data imply CD20^+^ melanoma cells as the major driver of melanoma progression, we here firstly report complete remission of melanoma upon targeting of the CD20^+^ subset of melanoma cells by the CD20-specific therapeutic antibody rituximab in off-label use in a patient with advanced metastatic melanoma.

## RESULTS

### Case Report

The patient, a 74-aged Caucasian male, received a diagnosis of stage IIIB (AJCC) ulcerated, acro-lentiginous malignant melanoma on the left heel with a tumor thickness of 2.0 mm in May 2010. Surgical tumor excision was conducted with a 3 cm margin. Inguinal lymph nodes were infiltrated with tumor cells, whereas popliteal lymph nodes were found to be free of tumor cells by lymph node scintigraphy and sentinel lymph node biopsy. Tumor cells exhibited wild-type alleles of BRAF and c-Kit as revealed by RT-PCR. The patient received adjuvant therapy with interferon-alpha (3 mio. IU s.c. three times a week) from August to October 2010. Left inguinal lymphadenopathy enforced lymphadenectomy in November 2010 with extirpation of 5 lymph nodes; three of them with metastatic infiltrations, one of which extended beyond the lymph node capsule. In February 2011, the patient progressed into AJCC stage IV (M1a) with disseminated lymph node metastases and multiple widespread cutaneous and subcutaneous metastases. The patient's skin and lymph node metastases still progressed during two further cycles of therapy with dacarbazine (1 g/m^2^ every 4 weeks) combined with epifocal dinitrochlorobenzene (DNCB) treatment in increasing doses from 0.1% up to 2% once per week accordingly to Trcka et al [11]. The patient did not meet inclusion criteria of ongoing trials. In May 2011, the patient was enrolled in an off-label use of rituximab according to the treatment schedule Table 1. The procedure was reviewed and approved by the institutional “Tumor Review Board of the Center for Integrated Oncology (CIO)”, University Hospital of Cologne.

**Table 1:** Treatment schedule of melanoma lesions

	*day -82*		*day -62*	*day -28*	*day -12*		*day 1*		
	**target lesion**				**target lesion**				
	p.m.	i.l.n.m.			p.m.	i.l.n.m.			
**treatment**									
rituximab in plantar lesion							2×10 mg		
rituximab in popliteal lesion							3×10 mg		
rituximab in inguinal lesion									
dacarbazine			1g/m^2^	1g/m^2^					
**biopsy**				plantar lesion					
**CT and MRI scans**	n.a.	15 mm			4.7x2 cm	19 mm			
	*day 7*	*day 15*	*day 20*	*day 29*	*day 36*	*day 48*	*day80*		
							**target lesion**		**RECIST**
							p.m.	i.l.n.m.	
**treatment**									
rituximab in plantar lesion	1×10 mg	1×10 mg	1×10 mg	1×10 mg	1×40 mg	1×40 mg			
rituximab in popliteal lesion	2×10 mg	2×10 mg	2×10 mg	2×10 mg	2×40 mg	2×40 mg			
rituximab in inguinal lesion	1×10 mg	2×10 mg	2×10 mg	2×10 mg	2×40 mg	2×40 mg			
dacarbazine			1 g/m^2^			1 g/m^2^			
**biopsy**					plantar lesion				
**CT and MRI scans**							1.3×1.1 cm	1.2 cm	PR
	*day 101*	*day 129*	*day 187*	*day 194*			*day 228*		
							**target lesion**		**RECIST**
							p.m.	i.l.n.m.	
**treatment**									
rituximab i.v.	187.5mg/m^2^	375mg/m^2^							
rituximab in plantar lesion									
rituximab in popliteal lesion									
rituximab in inguinal lesion			2×40 mg	2×40 mg					
dacarbazine	1 g/m^2^	1 g/m^2^							
**biopsy**								surgery	
**CT and MRI scans**							CR	0.9 cm	PR

p.m., popliteal metastasis; i.l.n.m., inguinal lymph node metastasis; n.a., not applied; PR, partial remission; CR, complete remission.

### Therapeutic anti-CD20 antibody injections

**Figure 1:**
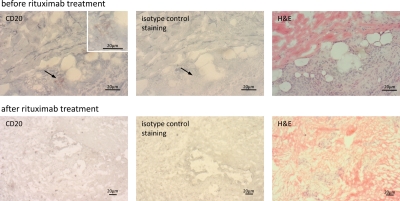
Rituximab treatment reduced the number of CD20^+^ melanoma cells in treated metastases Metastatic melanoma lesions harbor CD20^+^ melanoma cells. Cryostat sections of a cutaneous metastatic melanoma lesion prior and after rituximab treatment were stained for CD20 and visualized by peroxidase-AEC reaction. The biopsy after rituximab treatment was taken from the same melanoma lesion but distant from first biopsy. Staining with an isotype matched antibody of irrelevant specificity served as control. Hematoxylin-eosin (H&E) staining shows the histology. Of note, rituximab binding does not interfere with staining by the anti-CD20 antibody used in this analysis.

A cutaneous melanoma lesion from the left heel was subjected to immunohistological screening for CD20^+^ melanoma cells. As shown in Fig. 1, a minority of cells in the melanoma lesion expressed CD20 which represented about 2% of melanoma cells. The patient was subjected to off-label therapy with intra-lesional rituximab injections after informed consent. Treatment started with rituximab injections at a total dose of 20 mg into a cutaneous metastasis at the left heel and 30 mg into metastases in the popliteal region (for treatment schedule see Table 1). Since no toxicity was observed, the same lesions were treated again at day 7 together with an additional inguinal lesion, each lesion with a total dose of 10 – 20 mg rituximab. The same procedure was repeated at day 15, 20 and 29. At day 36, injected dose of rituximab was increased to 40 – 80 mg per lesion and repeated 12 days later. The ongoing treatment with dacarbazine (1 g/m^2^) every four weeks was continued and administered on days 20 and 48. Aside from minimal swelling due to the injection volume, no local or systemic toxicity was observed. Rituximab was administered by i.v. injection in a dose of 187.5 mg/m^2^ at day 101 and in a dose of 375 mg/m^2^ at day 129 followed by local injections into the inguinal lesion at day 187 and day 194. The remaining inguinal lesion was removed by surgery at day 228.

### Clinical response and evaluations

**Figure 2:**
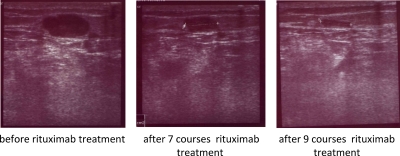
Sonographic recording of the popliteal left metastasis during rituximab therapy The metastasis substantially shrinks during therapy from approximately 722 mm^2^ to 135 mm^2^, i.e., a reduction by 80%; note that the blood vessel immediately under the metastasis showed nearly the same diameter in these projections.

**Figure 3:**
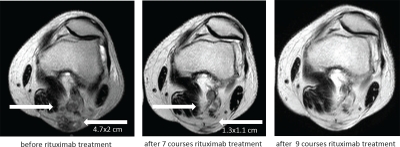
Complete regression of the metastases in the left popliteal region during rituximab therapy Popliteal metastases were screened by MRI before the start of rituximab treatment (day -28) and after 7 and 9 courses of local treatment. The popliteal bulk of metastases measured 4.7 × 2 cm before therapy and regressed to 1.3 × 1.1 cm and disappeared after 9 courses of treatment (right arrow). Left arrow indicates a solitary metastasis which completely disappeared after 7 courses. Complete remission was stable.

**Figure 4:**
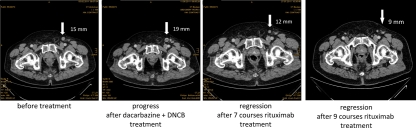
Partial regression of the inguinal metastasis during rituximab therapy CT scans of the inguinal region before the start of any treatment (day -82), dacarbazine plus DNCB treatment (day -12), and after 7 and 9 courses of rituximab injections. The inguinal metastasis progressed during chemotherapy from 15 mm to 19 mm in diameter; rituximab treatment produced substantially regression to 12 mm after 7 courses and to 9 mm after 9 courses of treatment, i.e., within 24 weeks. The solitary metastasis was stable and removed by surgery.

Cutaneous melanoma lesions and metastases continuously progressed during treatment with dacarbazine and DNCB. After 7 courses of rituximab injections, however, treated metastases had substantially regressed or were no longer detectable (Table 1). Histological screening of a treated lesion in far distance from the first biopsy confirmed morphological destruction of the melanoma tissue; no CD20^+^ melanoma cells were detected (Fig. 1). Interestingly, no substantial infiltration by immune cells occurred during treatment. Sonographic recording confirmed continuous regression of a left popliteal metastasis from approximately 38 × 19 mm to 15 × 9 mm in diameter within the course of 7 rituximab treatments (Fig. 2). Regression of melanoma lesions was furthermore objectified by MRI scan as exemplarily shown in Fig. 3. Both popliteal metastases regressed during treatment and complete remission was achieved within 7 months of rituximab therapy. Although they had not received intra-tumoral injections, the inguinal metastasis regressed during therapy as well (Fig. 4). While the metastasis progressed during chemotherapy from 15 mm to 19 mm in diameter within 3 months, they regressed to 9 mm as revealed by CT scans and was removed by surgery 7 months after first rituximab treatment (Fig. 4).

**Figure 5:**
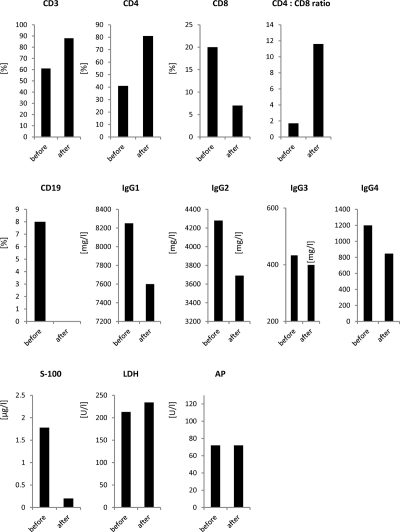
Laboratory parameters Serum and blood cell parameters were determined 6 weeks before and after 6 weeks of rituximab treatment. Serum melanoma marker S-100 and the enzymes lactate dehydrogenase (LDH) and alkaline phosphatase (AP) as well as the serum immunoglobulin-G (IgG) subsets were determined. The respective lymphocyte subsets of B cells (CD19^+^) and of T cells (CD3^+^, CD4^+^, CD8^+^) were determined by flow cytometry.

Along with the regression of metastatic melanoma lesions, serum S-100, a marker for melanoma progression, declined during rituximab therapy from 1.78 μg/l to near physiological levels (0.23 – 0.11 μg/l) (Fig. 5). Other serum enzymes including LDH and AP were in the physiological range. Recording of peripheral blood immune cells revealed complete depletion of CD19^+^ B cells, accompanied by decline in gamma-globulin levels, which was most likely due to rituximab targeting healthy B cells. CD4:CD8 T cell ratio was increased with increase in CD4^+^ helper T cells to 81% and decrease in effector CD8^+^ T cells to 7%. Before rituximab treatment the panel of serum cytokines indicated a predominant T_H_2 immune status which changed to a more pro-inflammatory T_H_1 cytokine signature at day 82 after rituximab treatment indicated by increase in IFN-γ, TNF-α, and other cytokines including IL-12 (Table 2). The elevated number of CD4^+^ helper T cells was accompanied by increased serum levels of IL-1β, IL-9, IL-10 and eotaxin pointing on their increased functional activity. The anti-angiogenic factor CXCL10 was additionally increased after rituximab treatment which together with a decreased VEGF level likely contributes to the shrinkage of the melanoma lesions.

**Table 2:** Serum cytokine profile before and after rituximab treatment

Cytokine	day –28 [pg/ml]^1^	day 82 [pg/ml]^1^	change
**IL-1b**	<1.91	20.82 ± 7.78	**+++**
**IL-1RA**	314.04 ± 11.67	772.17 ± 6.36	**++**
**IL-2**	<1.03	<1.03	**o**
**IL-4**	5.17 ± 9.90	31.09 ± 18.74	**+**
**IL-5**	<1.82	<1.82	**o**
**IL-6**	37.58 ± 28.64	18.84 ± 3.54	**o**
**IL-7**	16.47 ± 7.07	15.43 ± 2.83	**o**
**IL-8**	28.43 ± 26.16	18.29 ± 3.18	**o**
**IL-9**	23.94 ± 3.18	71.08 ± 2.41	**++**
**IL-10**	<1.53	30.19 ± 22.63	**++**
**IL-12(p70)**	31.97 ± 9.90	99.51 ± 9.19	**++**
**IL-13**	<2.22	21.77 ± 1.77	**+++**
**IL-15**	<1.69	<1.69	**o**
**IL-17**	15.85 ± 4.95	13.65 ± 2.83	**o**
**Eotaxin**	<6.51	85.27 ± 5.30	**+++**
**bFGF**	24.08 ± 7.42	25.41 ± 0.71	**o**
**G-CSF**	57.64 ± 3.18	120.95 ± 1.06	**++**
**GM-CSF**	43.31 ± 54.45	130.24 ± 12.73	**++**
**IFN-g**	175.83 ± 11.31	1000.62 ± 19.09	**+++**
**CXCL10**	635.84 ± 172.89	1787.78 ± 8.49	**++**
**CCL2**	32.78 ± 62.93	85.83 ± 10.61	**o**
**CCL3**	11.96 ± 20.15	16.71 ± 0.71	**o**
**CCL4**	129.09 ± 250.32	217.72 ± 14.14	**o**
**PDGF-bb**	8051.16 ± 873.28	6977.90 ± 57.28	**-**
**TNF-a**	<19.18	153.91 ± 8.49	**+++**
**VEGF**	144.04 ± 47.73	87.91 ± 1.77	**-**

1 mean +/− SD; < below detection level; + increase < 2fold; ++ increase ≥ 2fold; +++ increase ≥ 5fold; - decrease < 2fold; - - decrease ≥ 2fold; - - - decrease ≥ 5fold; o no significant change

## DISCUSSION

We report that local treatment with the therapeutic anti-CD20 antibody rituximab produced complete regression of all melanoma metastases with the exception of one lesion which showed partial but stable regression. The observation is unexpected since the melanoma metastases were continuously progressing during chemotherapy while rituximab therapy targeted CD20^+^ melanoma cells which comprised about 2% of cells in the lesions whereas the majority of melanoma cells lack CD20. After the elimination of CD20^+^ melanoma cells, notably, the number of CD20^−^ melanoma cells declined accompanied by a decrease in the S-100 serum marker to near physiological levels.

It was unexpected that the very low doses of lesionally applied rituximab would result in clinically evident tumor regression. Indeed, the injected doses of 10 − 40 mg rituximab per lesion once a week were several orders of magnitude below doses used in current lymphoma therapy in which rituximab is systemically administered [12]. Subcutaneous drug injections, almost produce some systemic leachate, even when the drug is administered at low doses, which explains tumor shrinkage at remote sites. The conclusion is sustained by the fact that locally injected rituximab was sufficient to clear healthy CD20^+^ B cells from the peripheral blood circulation in long-term as indicated by flow cytometric decline in B cell counts and the gradual decline in serum gamma-globulin levels. Loss of regulatory B cells and concomitant augmented T cell immunity, however, may contribute to but are unlikely to be entirely responsible for the effect since tumor shrinkage was observed before decline in blood B cell counts. B cell lymphopenia as a side effect of targeting CD20^+^ melanoma cells did not produce recurrent infections in the patient and we expect B cell repopulation after discontinuation of treatment as known in rituximab lymphoma therapy [12].

Melanoma regression is likely accompanied by a concerted action of various anti-tumor mechanisms including a cellular immune response and vascular re-modeling. The assumption is sustained by our observation that serum cytokine levels changed from a pronounced T_H_2 signature before rituximab therapy to a more pro-inflammatory T_H_1 signature with a nearly 8fold increase in IFN-γ and TNF-α levels indicating a strong induction of an adaptive immune cell response. Concomitantly, the functional activity of CD4^+^ helper T cells was improved indicated by increased serum levels of IL-1β, IL-9, IL-10 and eotaxin. We did not observe, however, auto-immune destruction of melanocytes resulting in vitilgo which is thought to be a side effect of anti-melanoma T cell response and is now discussed to play a role in supporting T cell responses to melanoma (13). Increased IL-12, on the other hand, contributes to boost the innate immune response while the increase of the anti-angiogenic factor CXCL10 together with a decreased VEGF level counteracts neo-angiogenesis and thereby likely contributes to the observed shrinkage of the melanoma lesions.

The therapeutic effect, however, is unlikely due to the concomitant dacarbazine therapy since melanoma lesions continuously progressed for 3 months during that treatment while stopped progression and turned into regression after the rituximab administration. Low dose dacarbazine treatment, however, may potentiate the effects of rituximab to some extent, for instance by sensitizing tumor cells which would otherwise survive therapy. Whether this or other pre-treatment regimens would further increase the anti-melanoma activity of rituximab remains to be resolved.

Whereas the cancer stem cell concept has thus far been based on pre-clinical models, the present data provide the first clinical evidence for a hierarchical tumor cell organization in an established melanoma lesion, in particular the predominance of CD20^+^ melanoma cells as the “master cell” required for maintaining and promoting an established melanoma lesion. The crucial feature of the therapeutic approach is that the minority of CD20^+^ melanoma cells needs to be therapeutically targeted to produce regression of the melanoma lesion as a whole. Recent data from our group [9] based on a pre-clinical transplantation mouse model provide strong evidence that T cell targeted elimination of the minority of CD20^+^ melanoma cells prohibit melanoma progression resulting in the eradication of the disease whereas the mass of cancer cells is not therapeutically targeted. Moreover, mathematical models which simulate the effect of targeting different tumor subset cells [8] support our observation that elimination of tumor-maintaining “master cells” is both required and sufficient to induce tumor regression, whereas increase in the death rate or decrease in the production of mature tumor cells may be auxiliary but inadequate to eradicate progressively growing cancer lesions when used alone. The delayed onset of tumor shrinkage after rituximab therapy is expected in this context. These models, however, do so far not take the plasticity of cancer cells into account, meaning that differentiated tumor stem cells may acquire stem cell properties when gaining access to the stem cell niche (14). Consequently, anti-stem cell therapy needs to be repetitively applied to eliminate those reverting cells and provides the rationale to administer rituximab repetitively over a longer period of time in our patient.

The same melanoma subset cells may be targeted by other therapeutic anti-CD20 antibodies like ofatumumab or the next generation antibody GA-101 which possibly show more potent in the therapy of chronic lymphocytic leukemia (15). Instead of antibodies, CD20-specific cytolytic T cells can be adoptively transferred which were engineered by expression of a recombinant T cell receptor or a chimeric antigen receptor; the latter was recently explored in a pre-clinical model [9]. Unlike cell-based therapies, antibody-mediated therapy has the benefit that antibody doses can be controlled in serum levels and are cleared with a defined half-life which is crucial in case of adverse events. Drug discovery approaches to target crucial signaling pathways in cancer stem cells including the Notch, Hedgehog and Wnt signaling pathway are currently explored at various levels (16). Complete response of chemotherapy-resistant melanoma highlights the potency of selectively eliminating tumor sustaining cells and warrants exploration of a greater patient cohort.

## MATERIALS AND METHODS

### General laboratory statement

Research sample processing, freezing, and laboratory analyses were performed in the CIO Studies Laboratory at the University Hospital of Cologne which operates under principles of Good Laboratory Practice with established SOP's and/or protocols for sample receipt, processing, freezing, and analysis.

### Immunohistochemical staining of biopsies

A cutaneous metastasis of the heel was excised in total, shock-frozen in liquid nitrogen, embedded in Tissue Tek^®^ Cryomolds™ containing O.C.T.™ compound (Sakura Finetek, Zoeterwoude, The Netherlands) and stored at −80 °C. Cryostat microtome 5 μm sections were fixed in ice-cold acetone and re-hydrated in PBS. Endogenous peroxidase activity was blocked with 3% (v/v) H_2_O_2_ for 10 minutes followed by blocking of unspecific binding sites with 10% (v/v) goat serum for 30 minutes. Slides were incubated with the monoclonal mouse anti-human CD20 antibody L26 (Abcam, Cambridge, UK) (5 μg/ml) for 1 hour, washed thrice in PBS and incubated with the horse radish peroxidase-conjugated goat anti-mouse-IgG antibody sc2005 (Santa Cruz Biotechnology, Santa Cruz, CA) (5 μg/ml) for 1 hour at room temperature. Incubation with a secondary antibody of irrelevant specificity served as isotype control. Labeled cells were detected by AEC substrate; cells were counterstained with hematoxylin.

### Serum cytokines

The concentration of a panel of 27 cytokines and chemokines in patient sera was determined using the “Bio-Plex Pro Human Cytokine 27-Plex Panel” (Bio-Rad, Munich, Germany) and the “Luminex Reader” according to the manufacturer's instructions.

### Study procedures

Cutaneous melanoma metastases on the lateral part of the left ankle, subcutaneous metastases in the left dorsal popliteal region and in the left inguinal region were treated by intra-lesional rituximab injections (10 mg in 4 ml NaCl 0.9%). Treated areas were first disinfected and anesthetized with a 2% (v/v) xylocaine injection. Rituximab doses and the treatment schedule are summarized in Table 1. No treatment related side effects occurred. Ongoing i.v. dacarbazine (1 g/m^2^ in 1L NaCl 0.9%) treatment was repeated at monthly intervals. Pre-medication was performed with granisetron (1 mg) and dexamethason (4 mg). Blood and serum samples were analyzed in the accredited Central Laboratory at the University Hospital Cologne.
